# Perceived social support on objective measured sedentary behavior of stroke patients: the mediating role of exercise self-efficacy

**DOI:** 10.3389/fpsyg.2024.1444214

**Published:** 2024-09-25

**Authors:** Jianing Wei, Fanjiayi Yang, Xiaofang Dong

**Affiliations:** ^1^School of Nursing and Health, Zhengzhou University, Zhengzhou, China; ^2^Department of Neurology, First Affiliated Hospital of Zhengzhou University, Zhengzhou, China

**Keywords:** stroke, sedentary behavior, social support, self-efficacy, mediation

## Abstract

**Introduction:**

As stroke patients may have impaired motor function, there may have increased sedentary behavior. Factors associated with sedentary behavior need to be explored to develop targeted interventions. Although studies explore the effects of exercise self-efficacy and perceived social support on sedentary behavior, the relationship is not clear. The aim of this study was to explore the mediating effect of exercise self-efficacy on perceived social support and objective measured sedentary behavior in stroke patients.

**Methods:**

The random sampling method was adopted to select 275 stroke patients from the neurology department of a third-grade hospital of Henan Province from June 2023 to March 2024 in a cross-sectional descriptive study. A general data questionnaire, exercise self-efficacy scale, perceived social support scale, and ActiGraph GT3X accelerometer were used to investigate. The structural equation model was used to analyze the mediating effect of exercise self-efficacy between perceived social support and sedentary behavior.

**Results:**

The mean sedentary behavior time in stroke patients in this study was (479.65 ± 112.65) min, the mean score of perceived social support was (47.53 ± 17.16), and the mean score of exercise self-efficacy was (24.19 ± 6.25). The correlation analysis revealed that, the sedentary behavior of stroke patients was negatively correlated with exercise self-efficacy and perceived social support. The results of the mediation analysis showed that exercise self-efficacy played a partial mediating role between perceived social support and sedentary behavior.

**Conclusion:**

Perceived social support and exercise self-efficacy were influential factors in sedentary behavior. Moreover, the impact of perceived social support on sedentary behavior was partially mediated by exercise self-efficacy. Therefore, to decrease the sedentary behavior, it is crucial to improve the level of perceived social support in stroke patients. Patients with less perceived social support can improve their exercise self-efficacy and thus perceive more social support.

## Introduction

Stroke is a prevalent chronic illness that poses a severe threat to human health, which is characterized by high rates of incidence, recurrence, mortality, disability, and disease burden. Globally, stroke is currently the second leading cause of death and long-term disability (Tsao et al., 2023), with more than 400,000 survivors living with complications (Krueger et al., 2015).

Stroke affects 1,115 individuals per 100,000 in China, with a mortality rate of 115 per 100,000, and over 2 million new cases are reported annually. Nearly 50% of stroke patients exhibit motor dysfunction after a stroke (Einstad et al., 2021). The incidence of stroke is significantly affected by a multitude of factors, encompassing behavioral, psychological, and socio-environmental elements. Among these, behavioral risk factors, notably sedentary behavior, contribute substantially to the rising prevalence of chronic diseases (O'Donnell et al., 2010).

Sedentary behavior (SB) is recognized behavioral factors critical for health (Sabia et al., 2022). Sedentary behavior, also known as static behavior, refers to the consumption of 1.5 metabolic equivalents (METs) in any awake state, including all sitting, reclining or lying, using electronic devices, writing, reading, talking and driving (Tremblay et al., 2017). SB causes many health problems and has been shown to be independently associated with cardiovascular-related mortality (Bull et al., 2020), disease morbidity (Biswas et al., 2015), hospitalization rates (Biswas et al., 2015), metabolic syndrome (Palmer et al., 2019), diabetes (Bull et al., 2020), cognitive function (Falck et al., 2017), and poorer mental health (Hallgren et al., 2018). In addition, stroke patients are the least active population of all cardiovascular and cerebrovascular patients (Ezeugwu and Manns, 2017), and about three-quarters of their waking time is spending in SB (English et al., 2016). Thus, SB in stroke patients should be valued.

Reduction and interruption of SB can improve the health indicators such as waist circumference, blood glucose level, insulin level, and body mass index, which is of positive significance for improving the quality of life (Healy et al., 2015). Current guidelines state that adults should spend < 8 h of SB per day (Piercy and Troiano, 2018). However, existing studies have found that SB in stroke patients does not meet the guideline requirements. Scholars Sjöholm et al. (2014) and Barrett et al. (2018) investigated hospitalized stroke patients and found that 74% and 86.9% of their daily time were sedentary, respectively. Wondergem et al. (2019) found that stroke patients spend an average of 9.3 h on SB per day. All the above studies show that stroke patients have too long SB, how to decrease the level of SB in stroke patients is the key. However, stroke survivors have many problems (Hamre et al., 2021), such as limbs disturbance, which makes it difficult for patients to complete SB recommended by the guidelines. Stroke prevention and treatment are facing bottlenecks, so it is crucial to explore how to reduce the sedentary behavior of stroke patients.

Previous studies have shown that various factors, including perceived social support (Northcott et al., 2016) and exercise self-efficacy (Wen et al., 2018), play a significant role in influencing the SB of patients who had a stroke. Perceived social support is a significant impact factor on health behavior (Blanton et al., 2019). It is widely recognized that social support constitutes an interactive process that encompasses both the provision and receipt of assistance. Crucially, for social support to be effective, it must be perceived as such by the individuals receiving it (Northcott et al., 2016). Studies (Kruithof et al., 2013; Holder et al., 2023) have shown that individuals who feel encouraged, understood, and supported by their social networks are more inclined to adopt and maintain healthy behaviors, including physical activity. Hall et al. (2020)'s study highlights the willingness of stroke patients to reduce SB with appropriate support from caregivers and professionals, and the Choi et al. (2022) study found that stroke patients who were married and lived with their spouse were less likely to tend to have SB. It can be seen that perceived social support is an important influencing factor of SB in stroke patients. Therefore, we propose hypothesis (a), perceived social support is negatively associated with SB.

Psychological aspects, such as exercise self-efficacy, were reported to have an effect on exercise (Trost et al., 2002). Self-efficacy, a central tenet of social cognitive theory, refers to the “belief in one's capabilities to organize and execute the courses of action required to manage given situations” (Bandura, 1977). It has been reported that a high level of exercise self-efficacy can predict the start and sustenance of exercise over time in patients with stroke (Nicholson et al., 2014), while low self-efficacy is the strongest barrier to behavior change in stroke patients (Mirkarimi et al., 2017). Wondergem et al. (2019) found that patients with higher self-efficacy had shorter time spending on SB. After stroke, short exercise cannot make patients see the effect of physical rehabilitation, resulting in low exercise self-efficacy and the increase of SB. Therefore, we propose hypothesis (b), exercise self-efficacy is negatively associated with SB.

Social cognitive theory (SCT) (Bandura, 2001) was constructed in 1986 by American psychologist Bandura. SCT posits that behavior is shaped by a combination of individual and environmental factors, which in turn influence changes in health-related behaviors. In our research, sedentary behavior was regarded as a key behavioral factor. For stroke patients, a high level of perceived social support can bolster their confidence in resuming a normal life. Concurrently, companionship and support can modulate the patients' negative emotions, sustain a stable psychological state, and enhance their self-efficacy, thereby potentially reducing sedentary behavior. Consequently, it is justifiable to adopt SCT as the theoretical framework for our study. Although studies have been conducted to explore the link between self-efficacy and social support and SB, the mechanisms underlying these three variables are unclear and require further research. According to the SCT, we propose hypothesis (c), the perceived social support on SB was partly mediated by exercise self-efficacy.

In summary, SB was hypothesized to be related to exercise self-efficacy and perceived social support, and that exercise self-efficacy may be a potential mediator between perceived social support and SB. This study aimed to quantify the levels of SB, exercise self-efficacy and perceived social support in stroke patients and to explore the potential relationship between SB and exercise self-efficacy and perceived social support. The results would provide a theoretical basis for developing interventions to improve SB in such patients.

## Methods

### Design

A descriptive cross-sectional design was utilized in this study, which followed the guidelines of the Strengthening Reports of Observational Studies in Epidemiology (STROBE) for cross-sectional studies.

### Participants

In this study, the convenience sampling method was used to select the research subjects in the department of Neurology of the First Affiliated Hospital of Zhengzhou University. Data were collected over a period spanning from June 2023 to March 2024. Participants were eligible for the study if they met the following inclusion criteria: (a) diagnosis of stroke, (b) age 18 years or older, (c) absence of cognitive dysfunction, as evidenced by a Montreal Cognitive Assessment (MoCA) score of 26 or higher; (d) consent from patients and their families to wear a Actigraph GT3X accelerometer following discharge from the hospital. Subjects were excluded from the study if they: (a) suffered from severe heart failure, respiratory failure, cancer, or were in other terminal stages of illness; (b) did not wear the triaxial accelerometer for a minimum of 3 valid days per week.

### Questionnaire data collection

The self-report questionnaires, including demographic and clinical information, exercise self-efficacy scale and perceived social support scale were used to complete the data collection. Before data collection begins, uniform training was conducted to ensure homogeneous object selection and reduce sample selection errors. First, the patients were screened according to the admission cases, and then patients are comprehensively evaluated to select patients who meet the criteria. Guiding language were used during the design of the questionnaire to facilitate patients to understand the questionnaire requirements, informing participants of the purpose of the study and the right not to participate or to withdraw from the survey at any time. Before discharge, paper questionnaires were distributed on-site, and the participants were asked to complete the questionnaires according to their actual situations. The average time to complete the questionnaire was approximately 25 min. In the data entry, the form of two-person entry should be adopted, and the incomplete data should be removed to ensure the accuracy of the data. A total of 280 questionnaires were distributed, with 275 valid questionnaires, achieving a valid recovery rate of 98.21%.

### Tools

#### Demographic and clinical information

The demographic information included age, gender, place of residence, education, marital status, physical activity before stroke, work status, and household, etc. Meanwhile, health specifics like the Nutritional Risk Screening 2002 (NRS2002), stroke stage, and the modified Rankin scale were collected from electronic health records of the patients.

#### Perceived social support scale

The PSSS is an instrument to measure the individual's self-perception of multidimensional social support. The scale was developed by Zimet et al. (1990) in 1987. The 12-item scale is designed to evaluate the level of support an individual perceives from family, friends, and others. The highest score of this 7-point Likert-type scale is 84, and higher scores mean better perceived social support. The Cronbach's alpha value of PSSS is 0.92. This scale has been applied to patients who had a stroke in other studies (Dong et al., 2022; Wang et al., 2019).

#### Exercise self-efficacy scale

The Exercise Self-Efficacy Scale, a self-reported scale invented by Kroll et al. (2007), measures self-efficacy to perform exercise in spite of numerous difficult situations. The scale is designed to assess individuals' confidence in their ability to engage in exercise a minimum of three times per week despite potential challenges. It encompasses 10 items and employs a 4-point Likert scoring system, with scores ranging from 10 to 40 points. The Cronbach's a coefficient of the Chinese ESES scale was 0.879 (*P* < 0.01), and the split-half reliability coefficient of the scale was r = 0.858 (*P* < 0.01). The test-retest reliability was 0.657 (*P* < 0.01) (Dong et al., 2016).

### Accelerometry measurement

Participants were instructed to wear the ActiGraph GT3X (Pensacola, FL, USA) on the left wrist (if the left wrist was affected by stroke, the right wrist was selected) on the day of discharge and for 7 consecutive days at a time outside of any aquatic activity. They were set to record three axes of acceleration (e.g., vertical, anterior-posterior and medium-lateral) at 30 Hz (30 times per s) with data smoothed using 1 s epochs. When receiving the accelerometer, participants were shown and carefully instructed, verbally and in writing, on how to properly wear and handle the ActiGraph GT3X accelerometer.

Raw accelerometer data (activity counts) were downloaded via ActiLife software (v 6.13.4, ActiGraph, Pensacola, FL, USA) for processing. Requirements for participants' data to be included in the analyses implied 3 valid days out of the 7-day period, with ≥480 min of wear time each day (English et al., 2016; Troiano et al., 2008; Joseph et al., 2020), given that 3-day accelerometer data have proved valid and reliable when measuring free-living sedentary behavior (English et al., 2018; Hendrickx et al., 2019). In addition, accelerometer non-wear time was defined as periods of ≥90 min of consecutive zeroes and were not included in the analysis (Choi et al., 2011). In agreement with previous research work about actigraphy in stroke population and elders (English et al., 2016; Troiano et al., 2008; Choi et al., 2011), Freedson et al. (1998)'s cut-points were applied to the data to classify SB intensity as ≤ 99 counts/min. Participants were excluded from the analysis if they did not meet valid wear criteria (Freedson et al., 1998).

### Ethical considerations

The study protocol was approved by the Ethics Committee of the First Affiliated Hospital of Zhengzhou University (2022-KY-1157-002). The study followed the principles of anonymity and confidentiality, and informed consent was obtained from the participants.

### Data analysis

The SPSSAU software V.21was used to analyze the data. Count data were described by frequency and composition ratio, whereas measurement data were described by $\bar{\text{x}}$ ± SD and compared between groups using independent samples *t*-test and one-way ANOVA. The measurement data that did not conform to a normal distribution were described by median and quartiles, and the Mann–Whitney U test and Kruskal–Wallis H test were used for comparison between groups. Pearson correlation analysis was used to analyze the interrelationships between the three variables. The pathways of action among perceived social support, exercise self-efficacy, and sedentary behavior were analyzed using SPSSAU, and indirect effects were estimated by the bootstrap resampling method with 5,000 replicate samples, and 95% confidence intervals (CI) were calculated. A mediating effect was considered significant if the 95% CI did not include zero. *P* < 0.05 (two-sided test) was considered statistically significant.

## Results

### Participant characteristics

A total of 275 participants successfully completed the data collection, resulting in a questionnaire response rate of 98.21%, as outlined in Table 1. The demographic composition of the participants was primarily male (64.36%), with a significant majority being married (81.09%), over half were unemployed (56.73%), and most mRS ratings were rated at level 1 (50.18%). In addition, participants had the most exercise once a week before the illness (45.45%), and most of them were in the acute phase of stroke (65.45). Detailed information of the sample population can be found in Table 1.

**Table 1:** Characteristics of the sample (*N* = 275).

**Variable**		**N (%)**	***Z/H***	***P***
Gender	Male	188 (64.36)	−0.006^a^	0.995
	Female	87 (31.64)		
Age	18–64	166 (60.36)	−0.787^a^	0.431
	≥65	109 (39.64)		
Place of residence	Rural area	134 (48.73)	−0.820^a^	0.412
	Town	141 (51.27)		
Education	Elementary school and below	95 (34.55)	9.814^b^	0.020
	Junior high school	116 (42.18 )		
	Senior high school	57 (20.73 )		
	College degree and above	7 (2.55)		
Marital status	Divorced or widowed	52 (18.91)	−5.011^a^	< 0.001
	Married	223 (81.09)		
Pre-stroke exercise	>5 times/week	76 (27.64)	0.973^b^	0.808
	4 or 5 times/week	29 (10.55)		
	2 or 3 times/week	45 (16.36)		
	≤ 1 times/week	125 (45.45)		
Employment status	Employed	119 (43.27)	−2.717^a^	0.007
	Not in workforce	156 (56.73)		
Household	Poor	25 (9.09)	0.849^b^	0.654
	Average	231 (84.00)		
	Good	19 (6.91)		
Stroke stage	Acute speriod	180 (65.45)	1.904^b^	0.386
	Recovery period	46 (16.73)		
	After-effects period	49 (17.82)		
NRS2002	Nutritional risks	141 (51.27)	−4.894^a^	< 0.001
	No nutritional risks	134 (48.73)		
mRS grade	0	30 (10.91)	12.364^b^	0.030
	1	138 (50.18)		
	2	51 (18.55)		
	3	29 (10.55)		
	4	22 (8.00)		
	5	5 (1.82)		

NRS2002, Nutritional risk screening 2002. ^a^*H*-value, ^b^*Z*-value.

### Correlation analysis between variables

The results of this study showed that all three variables, SB, PSSS, and ESES were correlated with each other. The mean SB time in stroke patients in this study was (479.65 ± 112.65) minutes, the average effective wear duration was (1,026.42 ± 165.85) minutes (see in Supplementary Table S1), while the mean score of PSSS was (47.53 ± 17.16), and the mean score of ESES was (24.19 ± 6.25), as displayed in Table 2. The correlation analysis is shown in Table 2, where SB was negatively correlated with both PSSS (r = −0.801, *P* < 0.01) and ESES (r = −0.573, *P* < 0.01).

**Table 2:** Correlation analysis of SB, PSSS, and ESES.

	**$\bar{\text{x}}$ ±SD**	**SB**	**PSSS**	**ESES**
SB^a^	479.65 ± 112.65	1		
PSSS	47.53 ± 17.16	−0.801^**^	1	
ESES	24.19 ± 6.25	−0.573^**^	0.508^**^	1

SB, Sedentary behavior; PSSS, Perceived Social Support Scale; ESES, Exercise Self-efficacy Scale. ^a^min. ^**^P < 0.01.

### Intermediary analysis

**Figure 1:**
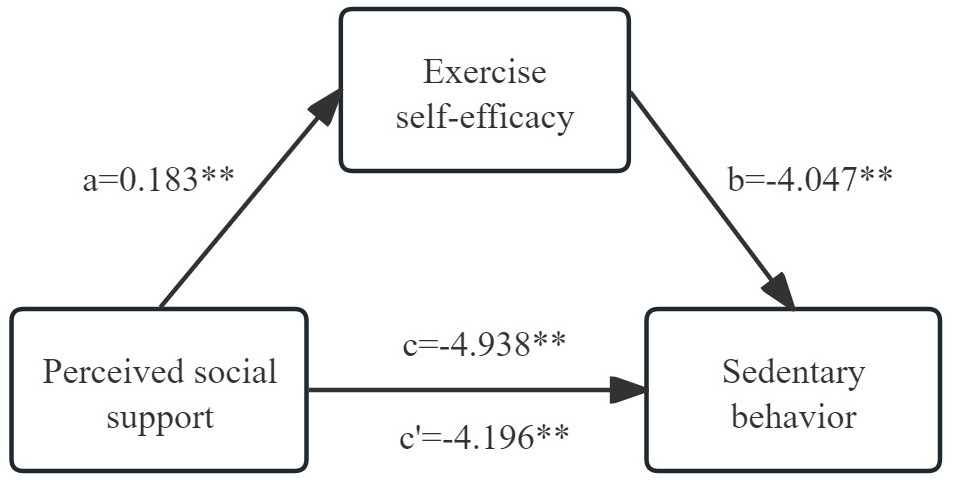
Mediating effect of exercise self-efficacy between perceived social support and sedentary behavior. a, effect of perceived social support on self-efficacy; b, effect of self-efficacy on sedentary behavior; c, total effect of perceived social support on sedentary behavior; c', direct effect of perceived social support on sedentary behavior. **p < 0.01.

Building on our initial findings from the correlation analysis, we next performed mediation analysis to explore the potential association underlying the relationship between a stroke patient's SB, PSSS, and ESES. PSSS was set as the independent variable, with ESES as the mediating variable and SB as the dependent variable, and path analysis was used to fit the hypothetical model. The model fitting results revealed that the chi-squared degrees of freedom ratio showed a good model fit, with the chi-square freedom ratio (χ^2^/df) = 0.000, the fitness index (GFI) = 0.966, the benchmarked fitness index (NFI) = 0.952, the comparative fitness index (CFI) = 0.951, and the asymptotic residual mean square and root square (RMSEA) = 0.000, as detailed in Table 3 and Figure 1.

**Table 3:** Fitting indicators and evaluation criteria.

**Fit index**	***χ2*/*df***	**GFI**	**RMSEA**	**NFI**	**IFI**	**CFI**
Evaluation criteria	< 3	>0.9	< 0.10	>0.9	>0.9	>0.9
Index value	0.000	0.966	0.000	0.952	0.952	0.951

χ^2^/df, Chi-square/degree of freedom; GFI, goodness-of-fit index; RMSEA, root mean square error of approximation; NFI, normed fit index; IFI, incremental fit index; CFI, comparative fit index.

We controlled for some factors (such as education, marital status, employment status, NRS2002 and mRS) which are considered important predictors that affect SB in patients with stroke. The results showed that PSSS predicted exercise self-efficacy (β = 0.183, t = 8.895, *p* < 0.001), and ESES significantly predicted SB (β = −4.047, t = −5.808, *p* < 0.001). Meanwhile, PSSS had a direct effect on SB was also significant (β = −4.196, t = −15.697, *p* < 0.001), but the comparison of the effect coefficients showed that the effect of PSSS on SB was diminished from −4.938 to −4.196, which meant that ESES partially mediated the effect between PSSS and SB, as shown in Table 4.

**Table 4:** Mediating effect of SB between ESES and PSSS.

	**SB**					**ESES**					**SB**				
	***B***	***SE***	***t***	***p***	β	***B***	***SE***	***t***	***p***	β	***B***	***SE***	***t***	***p***	β
Intercept	730.414^**^	31.353	23.297	0.000	–	17.119^**^	2.595	6.597	0.000	–	799.692^**^	31.910	25.061	0.000	–
Education	0.254	4.994	0.051	0.959	0.002	−0.132	0.413	−0.319	0.750	−0.017	−0.279	4.715	−0.059	0.953	−0.002
Marital status	−21.855^*^	10.631	−2.056	0.041	−0.076	−0.307	0.880	−0.349	0.727	−0.019	−23.099^*^	10.038	−2.301	0.022	−0.080
Employment status	−10.144	8.250	−1.230	0.220	−0.045	−0.088	0.683	−0.129	0.898	−0.007	−10.500	7.788	−1.348	0.179	−0.046
NRS2002	14.389	8.387	1.716	0.087	0.064	−0.528	0.694	−0.760	0.448	−0.042	12.253	7.926	1.546	0.123	0.054
mRS grade	10.938^**^	3.420	3.198	0.002	0.114	0.058	0.283	0.204	0.839	0.011	11.171^**^	3.229	3.460	0.001	0.116
PSSS	−4.938^**^	0.249	−19.843	0.000	−0.752	0.183^**^	0.021	8.895	0.000	0.503	−4.196^**^	0.267	−15.697	0.000	−0.639
ESES	–	–	–	–	–	–	–	–	–	–	−4.047^**^	0.697	−5.808	0.000	−0.225
*R^2^*	0.667					0.261					0.705				
Adjusted *R^2^*	0.660					0.244					0.697				
*F*	*F*_(6, 268)_ = 89.608, *p* = 0.000					*F*_(6, 268)_ = 15.739, *p* = 0.000					*F*_(7, 267)_ = 91.007, *p* = 0.000				

NRS2002, Nutritional risk screening 2002; SB, Sedentary behavior; PSSS, Perceived Social Support Scale; ESES, Exercise Self-efficacy Scale. β, standardized regression coefficient; SE, standard error; ^*^p < 0.05 ^**^p < 0.01.

## Discussion

Understanding the mediators of the relationship between PSSS and ESES is crucial for developing strategies to reduce SB in stroke patients. Previous studies have only explored the relationship between SB, PSSS, and ESES separately, but ESES has been neglected as a mediator. This study confirmed our hypothesis, revealing a negative association between PSSS and ESES and SB. More importantly, ESES partly mediated the relationship between PSSS and SB. Our findings provide directions for the development of interventions to manage SB in such patients.

We found that the daily SB in stroke patients was 479.65 ± 112.65 min, nearly 8 h a day, that is, higher levels of SB in stroke patients, which is consistent with previous findings (Sjöholm et al., 2014; Barrett et al., 2018). Some stroke patients have problems of limb dysfunction after illness, especially those with impaired lower limb function, with longer SB. This suggests that further research and focus on SB and its influencing factors in stroke patients are urgently needed to lay the basis for subsequent designation of SB-related interventions. In this study, the average PSSS score was 47.53 ± 17.16, which means in a medium social support state. In other words, the social support for stroke patients is generally at a low or moderate level. Our study also found that the degree of PSSS in stroke patients was negatively associated with SB (r = −0.801, *P* < 0.01), which is consistent with our study hypothesis. We speculate that the reason for this result is that most stroke patients will leave physical disorders, which will affect the normal activities, and the accompanying support from relatives and friends can help patients to actively participate in rehabilitation exercise programs and promote the occurrence of healthy behaviors such as physical activity (Pauly et al., 2021). And the importance of social support is indicated in the existing studies, a cross-sectional study (Wen et al., 2022) enrolled 133 hypertensive stroke patients 6 months after discharge in China have shown the perceptions of chronic illness resources received from healthcare teams, family and friends, and the community were positively correlated with health behavior. Brouwer-Goossensen et al. (2021) have found social support was a positive factor in health behavior change in interviews with 18 patients who had a stroke. Therefore, perceived social support is an important factor in reducing SB in patients with stroke.

Our study suggests a negatively correlation between ESES and SB in patients with stroke, that is, stroke patients with higher levels of ESES have lower levels of SB. A higher level of self-efficacy can effectively change some's perception of the disease and improve psychological adaptation and the enthusiasm to participate in rehabilitation (Dong et al., 2022). However, the patients in this study had a mean ESES score of 24.19 ± 6.25, with longer SB. Most importantly, we found that SB appeared to correlate with health behavior via two pathways, a direct pathway and an indirect pathway mediated by ESES. High level of PSSS can improve patients' confidence to increase physical activity, regulate negative emotions with companionship and communication, and increase self-efficacy. Meanwhile, influenced by PSSS, they are more willing to seek help from others when ESES is low, which also promotes the reduction of SB.

Overall, our study found that PSSS was not only directly and negatively associated with the SB of patients with stroke, but also further related to SB via ESES. This was consistent with the connotation of the SCT (Bandura, 1978). In other words, our findings provided practical support for the SCT. That's to say, PSSS may influence patients' ESES and, in turn, their SB. Additionally, a positive correlation between PSSS and ESES has been shown, in order to reduce SB in stroke patients, we can start with both PSSS and ESES.

### Strengths and limitations

The strengths of this study are as follows. Firstly, the main outcome of this paper, sedentary time, was measured by an objective accelerometer, which is more accurate and reliable than a questionnaire. Second, there have been many studies on the factors influencing sedentary behavior, which include the independent effects of exercise self-efficacy and social support. However, this study is the first time to consider the two together to verify the mediating role of exercise self-efficacy, which can be used as a breakthrough point in future intervention research to conduct a new form of intervention.

Nevertheless, the limitations of the study should be acknowledged. Firstly, owing to the cross-sectional design of this study and the application of mediation analysis, only correlations between the variables were demonstrated, while the causal relationships between perceived social support, exercise self-efficacy, and sedentary behavior could not be determined. A longitudinal study is needed to determine the causal relationship between the variables in the future. Secondly, this study used the convenience sampling technique, which may lead to some degree of bias. In future study planning, alternative sampling methods are recommended to reduce the possibility of bias. Thirdly, the subjects of this study came from one hospital, and could be sampled from multiple hospitals or communities in the future to increase the sample size of the study. Furthermore, SB in this study was affected by the accelerometer wear duration, although we considered the total wear duration and effective percentage, measurement bias should be considered in designing more rigorous experiments in future studies. Finally, most of the stroke patients included in this study were mild, which may cause some shift in the interpretation of the results, such as sedentary time may be less relative to severe patients.

### Relevance to future research direction

The present study further confirms the high level of SB in stroke patients. As SB affects the prognosis of stroke patients and significantly impacts their recovery, elucidating the factors that contribute to SB and exploring measures to reduce SB is essential in stroke patients. The results of this study suggest that PSSS can indirectly influence SB in stroke patients due to the mediating role of ESES. On the basis of this study, future studies can be carried out in the following directions. First, the subjects of this study are stroke patients, and there are many people with SB in real life, such as hypertension patients and diabetes patients, so future studies can explore whether the conclusions of this study are applicable in other populations. Secondly, there are many intervention programs for sedentary behavior, and this study proposes the mediation role of exercise self-efficacy, which can provide some basis for the development of future intervention methods. Moreover, this study is a cross-sectional study, and a future longitudinal study design can be adopted to explore the causal relationship among the three variables. Finally, most of the stroke patients included in this study were mild and severe patients were excluded, and patients with different disease severity should be included in the future to provide a more comprehensive outcome.

## Conclusion

In conclusion, this study shows that PSSS and ESES are key correlates of SB in stroke patients, and that after controlling for the effects of confounders, ESES partly mediates the relationship between PSSS and SB. This suggests that healthcare professionals should pay attention to the assessment of patient' ESES in their clinical work. Appropriate measures should be taken to increase patients' ESES, such as educating stroke patients about the disease, the disadvantages of SB and the benefits of increased physical activity. Encourage patients to actively participate in social life, face the disease with a positive attitude, and reduce negative emotions, which is important to reduce SB.

## Data Availability

The raw data supporting the conclusions of this article will be made available by the authors, without undue reservation.
