# Supplementary material for: Perceived social support on objective measured sedentary behavior of stroke patients: the mediating role of exercise self-efficacy
Source: Front Psychol. 2024 Sep 25;15:1444214. doi: 10.3389/fpsyg.2024.1444214 (PMC11461327; doi:10.3389/fpsyg.2024.1444214)
Supplement: Supplementary file 1 [file Table_1.docx]

Appendix 1

**Table S1 SB, the effective wearing time and the ratio**

|  | SB | Wearing Time | Ratio |
| --- | --- | --- | --- |
| x̄ ± SD | 479.65 ± 112.65 | 1026.42±165.85 | 47.93±13.75 |

**Abbreviations:**SB,Sedentary behaviour;
